# MafK accelerates *Salmonella* mucosal infection through caspase-3 activation

**DOI:** 10.18632/aging.203938

**Published:** 2022-03-08

**Authors:** Shiyao Xu, Guiqiu Hu, Di Wu, Xingchi Kan, Hisashi Oishi, Satoru Takahashi, Shoupeng Fu, Juxiong Liu, Chuan Zhang

**Affiliations:** 1 Department of Basic Veterinary Medicine, College of Veterinary Medicine, Jilin University, Changchun 130062, Jilin, China; 2 Department of Comparative and Experimental Medicine, Nagoya City University Graduate 24 School of Medical Sciences, Aichi 467-8601, Nagoya, Japan; 3 Institute of Basic Medical Sciences and Laboratory Animal Resource Center, University of Tsukuba, Ibaraki 305-8575, Tsukuba, Japan; 4 Department of Endocrinology and Metabolism, The Second Hospital of Jilin University, Changchun 130000, Jilin, China

**Keywords:** MafK, *Salmonella*, colitis, apoptosis

## Abstract

Gastrointestinal homeostasis is critical for maintaining host health, and is affected by many factors. A recent report showed that Musculoaponeurotic fibrosarcoma K (MafK) expression is increased in patients that have ulcerative colitis (UC). Even so, MafK’s significance in sustaining intestinal homeostasis has not been investigated. In this research, MafK overexpressing transgenic (MafK Tg) mice were found to be more susceptible to infection with *Salmonella* on the mucosa than the wild-type (WT) mice. Following *Salmonella* oral infection, MafK Tg mice suffered higher mortality and a lot more weight loss, damage to the intestines, and inflammation in the intestines than WT mice. MafK Tg mice were also unable to control *Salmonella* colonization and dissemination. *In vivo* data showed that increased MafK expression promoted epithelial cell apoptosis which was further confirmed by *in vitro* data. The rapid cleavage of caspase-3 in epithelial cells contributed to *Salmonella* dissemination and inflammation initiation. This study reveals that MafK participates in *Salmonella* pathogenesis acceleration by increasing caspase-3 activation.

## INTRODUCTION

The intestinal epithelium, as a barrier, is important for the human defense system. It can isolate the aseptic environment of the host from the intestinal environment with various microorganisms settled, and is accountable for absorbing nutrients, immunity and excretion. Intestinal epithelial cells (IECs) have a significant impact on defending against pathogen invasion through appropriate activation of their signaling pathway and moderating the proinflammatory response [1]. The formation of tight junction and secretion of mucus are examples of the barrier function. Proper barrier function is essential for maintaining intestinal homeostasis. Uncontrolled cellular proliferation at the crypt base might lead to tumorigenesis, while increased cell death at the villus tip might lead to the disintegration of the epithelial barrier with severe pathogen invasion and intestinal inflammation development, for instance, ulcerative colitis (UC) and Crohn’s disease (CD) [2]. Intestinal epithelial cells (IECs) provide a variety of functions in addition to acting a physical barrier; they also perform other functions that are essential for the maintenance of intestinal homeostasis in the gastrointestinal tract. Intermediates of metabolism in IECs could activate the complicated network of connection between diet, the microbiota and immune cells to maintain intestinal homeostasis, and to be a therapeutic target that is critical in the treatment of inflammation and metabolism-associated disorders [3]. The cross talk between IECs and intestinal microbiota are also essential for intestinal homeostasis [4]. The response of IECs to signals provided by commensal microbiome that plays a role in disease progression regulation, such as type 2 diabetes mellitus, stress and depression [5–7]. Breakdown of the homeostasis of IECs leads to intestinal disorder, to give an example inflammatory bowel disease (IBD). Under inflammatory conditions, the intestinal mucosa undergoes complex mechanisms including intestinal epithelial cell proliferation and apoptosis, restitution and differentiation. Therefore, understanding the immune protection mechanism of regulation is important for maintaining intestinal homeostasis. Bacterial gastrointestinal infections in animals and humans are frequently caused by *Salmonella* enterica serovar Typhimurium [8], and is often used as a useful model for the study of intestinal barrier function [9].

Musculoaponeurotic fibrosarcoma K (MafK) belongs to the small macrophage-activating factor (Maf) protein family, low-stringency cDNA library screening of the v-Maf gene first identified it [10]. Normally, sMafs form homodimers, which form heterodimers with Cnc proteins (P45NF-E2, Nrf1, Nrf2 and Nrf3) or Bach proteins (Bach1 and Bach2) [11]. The fine regulation of gene expression by homodimers and heterodimers of the MafK protein participates in the process of development, differentiation and tumorigenesis [12, 13]. Some studies have shown that quantitative control of the MafK protein level may significantly induce hematopoietic differentiation [14]. MafK is also critical to the differentiation of early mesoderm cells, mesenchymal cells, hematopoietic cells and nerve cells [15]. MafK overexpressing mice develop hyperglycemia resulting from impaired glucose-stimulated insulin secretion in adulthood. Overexpression of MafK in mice can damage endocrine development by regulating a large number of β-cell-related genes [16]. Studies have shown that embryos of mice with sMaf protein (MafF, MafG and MafK) gene deletion showed embryonic lethality and apoptosis of fetal hepatocytes [17]. However, the biological significance of MafK in the intestinal tract is uncertain. The purpose of this research aimed to elucidate the mechanism of MafK in regulating intestinal homeostasis especially its role in limiting intestinal pathogen invasion.

## MATERIALS AND METHODS

### Mice

The generations of rat Insulin 1 promoter-controlled transgenic mice that express MafK (MafK Tg) (ICR background) were donated by Medical Laboratory of Tsukuba University, Japan. ICR mice were purchased from Liaoning Changsheng biotechnology company (China) and used as wild type (WT) mice. All the mice were housed in individual ventilated cages system with normal diet. The Jilin University Animal Welfare and Research Ethics Committee gave its approval for the animal trials.

### *In vivo* infection

In this study, Mice aged 6-8 weeks that were sex matched were used. After cultured in LB overnight at 37° C, *Salmonella* SL1344 was transferred to 4 M NaCl medium that have a high osmosis, and incubated for 4–6 h until OD_600_=0.6. As previously stated, a model of *Salmonella*-induced colitis was constructed [1]. Be concise, each mouse was given Streptomycin in the amount of 20 mg. Then, 5×10^7^ colony-forming units (CFU) *Salmonella* was given to the mice orally, the log CFU per organ was measured. During the survival experiment, mice were given a dose of 1×10^8^ CFU *Salmonella* orally.

### Evaluation of intestinal histopathology

Tissue samples of cecum were collected on Day 2 p.i. and the feces in cecum were washed with cold PBS. Following that, cecum tissue samples were preserved in 4% methane for 24 h, embedded in paraffin after being dehydrated in ethanol. 5 μm slices were used in hematoxylin and eosin (H&E) or immunofluorescence. As previously mentioned, histopathology of the tissue was evaluated blindly [18].

### Cytokine measurements

The cecum tissues were washed free of feces, then the weighted tissues were mechanically homogenized in 4 volumes of cold PBS. The supernatants used for cytokine examination were obtained by homogenates centrifuged at 4° C (13000 g) for 30 min. Cytokines were quantified in cecum tissue using an ELISA kit as directed by the R&D manufacturer’s instructions.

### Myeloperoxidase (MPO) activity

Harvest cecum tissues (10 mg) were rinsed in PBS at 4° C, homogenized and resuspended in 4 volumes of MPO Assay buffer (Sigma-Aldrich), the supernatant was collected and used after centrifuging 10 min at 4° C 13000 g. The assay was carried out in accordance with the guidelines provided by the manufacturer.

### Immunofluorescence and immunohistochemistry

5 μm paraffinic sections of cecum tissue were deparaffinized by xylene twice, rehydrated by different gradient ethanol. Then cell perforation of the sections by 0.1% Triton-100, 15 minutes in 10 mM citrate buffer for antigen retrieval. And the sections were blocked in 5% donkey serum for 1h. For immunofluorescence, Rabbit-anti-ki67 (Santa Cruz), rabbit-anti-Cleaved Caspase-3 (Abcam) and rabbit-anti-claudin3 (Abcam) were used as primary antibody incubate overnight 4° C. Using Donkey polyclonal Secondary Antibody to Rabbit IgG - H&L (Alexa Fluor® 488) (Invitrogen) and Donkey polyclonal Secondary Antibody to Rabbit IgG - H&L (Alexa Fluor® 488) (Invitrogen) as secondary antibody incubate for 1 h. One-step TUNEL assay kit (KeyGEN Biotech) was used to examine apoptosis of intestinal epithelial cell. DAPI (Abcam) as a counter-stain for DNA was used to staining nucleus. The assay was carried out in accordance with the manufacturer guideline. The sections were storage at 4° C in the dark for microexamination. For immunohistochemistry, after the sections of cecum tissue being blocked, Endogenous Peroxidase blocking buffer incubated for 10 min, then PBS washed 3 times for 3 min every time. Unspecific staining blocker incubated for 10 min. Primary antibody Mucin 2 antibody (Santa Cruz), Ly-6G/Ly-6c and F4/80 (Biolegend) were incubated overnight at 4° C after remove the unspecific staining blocker. Then Biotin-labeled goat anti-mouse IgG (H+L) incubated for 10 min, PBS washed 3 times for 3 min every time. Streptavidin- peroxidase incubated for 10 min, PBS washed 3 times for 3 min every time. Using DAB Horseradish Peroxidase Color Development Kit (Maixin-Bio) to incubate with the guidelines provided by the manufacturer. Haematoxylin was used to stain nucleus.

### FITC-dextran assay

The assay was used to examine the *in vivo* intestinal permeability. First, 5×10^7^ CFU of *Salmonella* or PBS was orally administrated to MafK and WT mice after pretreated with Streptomycin. After 48 h, fast mice for 4 h prior to the assay, but free to water. Then 50μL blood samples were taken from the tails of mice to detect the pre-gavage fluorescence. FITC-dextran (Sigma-Aldrich) was given to the mice at a dose of 600 mg/kg orally. After gavage for the first mouse, 50μL blood samples were taken again from the tails of mice post-gavage. The blood samples were mixed with anticoagulant (Sigma-Aldrich) at a dose of 15% v/v, the plasma was then collected by centrifugation for 10 min at 4° C (5000 g). The samples were diluted 4 times or 10 times by PBS and keep from light at 4° C. Fluorescence of the samples was determined at 530 nm with excitation at 490 nm as described [19].

### Western blotting analysis

The tissues of cecum were cleaned by cold PBS, dried by filter paper and weighted. For total protein extraction, the tissues were mixed with cell lysis buffer (Beyotime) at a ratio of 1:4000 g/mL, and homogenized by high-throughput tissue grinders, then the supernatant was collected centrifuged for 10 min at 4° C (5000 g). BCA protein assay kit (Thermo) was used to quantify protein concentration. The Nuclear and Cytoplasmic Protein Extraction Kit (Beyotime Biotechnology) was used to extract nuclear and cytoplasmic protein from Caco-2 cells as specified by the manufacturer. SDS-PAGE electrophoresis was used to separate the proteins in the samples. The proteins were transferred from the gels to a 0.22 μm PVDF membrane (Millipore). According to the protein molecular weight, obtained the corresponding PVDF membrane bands. 5% skim milk (BD Difco) was used to block the PVDF membrane for 2 h. Anti-iNOS antibody (Abcam), Anti-Cox-2 antibody (Abcam), Anti-Claudin3 antibody (Abcam), Anti-Cleaved Caspase-3 antibody (Abcam), Anti-MafK (Abcam) and Anti-p65 antibody (Abcam), Anti-β-Tubulin antibody (Sungene Biotech) as primary antibodies were diluted by 5% bovine serum albumin (BSA) following the dilution ratio supply by the manufacturer and incubated on the PVDF membrane at 4° C overnight. Then the primary antibodies in the PVDF membrane were washed away by 1×TBST (Solarbio). Goat anti mouse IgG-HRP (Santa) and goat anti rabbit IgG-HRP (Santa) as second antibodies, 5% BSA was used to dilute the antibodies at a ratio 1:4000 and incubated on the PVDF membrane for 1 h. Protein bands image on PVDF membrane developed by ECL (Applygen Technologies).

### Real-time quantitative PCR

The TRI reagent (Sigma-Aldrich) was used to extract the RNA from cecum tissues and Caco-2 cells with the guidelines provided by the manufacturer. The total RNA was quantified by Thermo Scientific NanoDrop. Then reverse transcription of RNA to cDNA was taken by MMLV reverse-transcriptase (Promega). For real-time quantitative PCR (qPCR), using 2×LightCycler^®^ 480 SYBR Green I Master (Roche) as a one-component hot start reaction mix for qPCR master reagent, 10 μM primer of forward and reverse, cDNA template and ddH_2_O. The sequences of the primer were: claudin3, 5’- CCTAGGAACTGTCCAAGCCG-3’ and 5’-CCCGTTTCATGGTTTGCCTG-3’; occludin, 5’-CAGCCTTCTGCTTCATCG-3’ and 5’-GTCGGGTTCACTCCCATTA-3’; ZO-1, 5’-GACCTTGAGCAGCCGTCAT-3’ and 5’-CCGTAGGCGATGGTCATAGTT- 3’; GAPDH, 5’-CACCCCAGCAAGGACACTGAGCAAG-3’ and 5’-GGGGGTCTGGGATGGAAATTGTGAG-3’.

### MafK overexpression and interference

The *in vitro* assay was performed in Caco-2 cell line preserved by our laboratory. For MafK overexpression, coding sequence (CDS) of MafK gene was cloned into pcDNA3.1 as pcDNA3.1(+)-3×Flag-MafK (Geneppl technology, co, Ltd) as treatment group. And Caco-2 cells were transfected with an empty vector as control group. Lipofectamine 2000 was used to transfect Caco-2 cells with the guidelines provided by the manufacturer.

MafK gene interruption was also performed with Caco-2 cell line. GenePharma designed and synthesized the siRNA duplexes to interrupt MafK gene expression, the sequence was: 5’-GACTAATCCCAAACCGAATAA-3’ (MAFK-Homo-217). And scrambled duplexes RNA as control group. For siRNA transfections, the Lipofectamine 2000 protocol was followed.

### *In vitro* infection

Caco-2 cells were cultured in complete medium (DMEM, Gibco; 10% FBS, Invitrogen), and planted at a density of 5×10^5^ cells/well in 6-well dishes (Thermo Fisher, DM) at 37° C for overnight. For MafK overexpression, caco-2 cells were transfected with pcDNA3.1(+)-3×Flag-MafK and pcDNA3.1, while for MafK interruption, caco-2 cells were transfected with MAFK-Homo-217 and scrambled duplexes RNA at density of 70-80% confluent. After transfection, cells were cultured in complete medium for another 48 h. Then using PBS to wash cells for 3 times and fresh Opti-MEM medium (Gibco) was replaced. 3×10^7^ CFU/well *Salmonella* strain SL1344 were added in the medium and collected the samples after 1 h.

### Co-immunoprecipitation

Caco-2 cells overexpressing or disrupted with MafK were treated with *Salmonella* for 60 min, and then lysed in immunoprecipitation lysis buffer. Next anti-MafK antibody or anti-Flag antibody were used to recognize and immunoprecipitate the target proteins in the whole -cell lysates, were probed with rabbit anti-p65 antibodies using western blotting.

### Statistical analysis

Image processing by Adobe Photoshop CC 2017, Data statistics and histogram processing by GraphPad Prism 8, gray intensity analysis by ImageJ, The mean and standard error of the mean (SEM) are used to represent the data. The one-way ANOVA (Dunnett's t-test), two-tailed Student's t-test and Logrank test were analyzed by SPSS 24.0 to represent the differences. Compare with control group *p < 0.05 represented significant difference and **p < 0.01 represented extremely significant difference.

## RESULTS

### MafK increases the susceptibility to *Salmonella* mucosal infection

**Figure 1:**
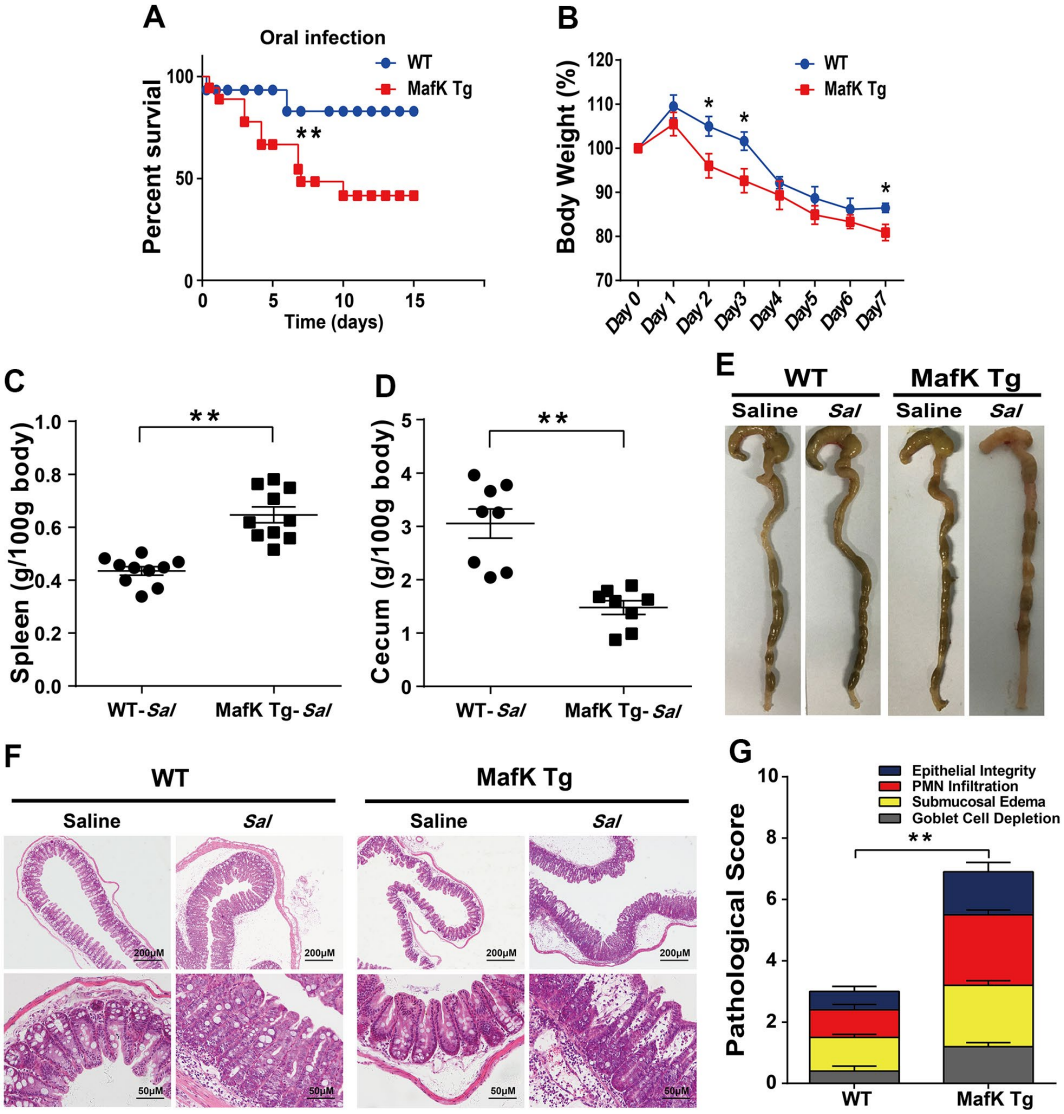
**MafK promotes *Salmonella* mucosal infection.** (**A**) Survival (n = 12 each group). (**B**) Body weight loss (n = 12 each group). (**C**) Weight of spleen (n = 10 each group) (**D**) weight of cecum (n = 8 each group). (**E**) Representative gross appearance. (**F**, **G**) Representative H&E staining of cecum tissue and pathological score. All data are shown as mean ± SEM. Student’s t-test was performed. Log-rank test was used for statistical analysis of animal mortality. Statistical significance is indicated by *p < 0.05, **p < 0.01.

To investigate the possibility that MafK is involved *Salmonella* infection, the mortality after being infected with *Salmonella in* WT and MafK Tg mice was examined Initially. WT and MafK Tg mice were oral administration with streptomycin before *Salmonella* infection. Mortality of MafK Tg mice was significantly higher than WT mice with an infective dose of *Salmonella* (1×10^8^ CFU) (Figure 1A). After *Salmonella* infected 10 day, 40% of the MafK Tg mice were died, whereas only 10% mice of the control group were died. MafK Tg mice lost much more body weight than WT mice (Figure 1B). To clarify the role of MafK in susceptibility to *Salmonella* mucosal infection, MafK Tg mice were infected with *Salmonella* at a lower dose (5×10^7^ CFU) in order to study their phenotype. The pathology of the cecum and spleen on Day 2 p.i. was measured. Splenomegaly was more severe in MafK Tg mice (Figure 1C) and had a considerable reduction in cecal weight when compared to WT mice (Figure 1D, 1E). Concurrently, according to the H&E staining of the cecum (Figure 1F) and colon (Supplementary Figure 3A), we validated the clinical assessments by histological examination. Histological evaluation of the cecum in MafK Tg mice revealed that the extent of epithelial damage, inflammatory cell infiltration, submucosal edema and goblet cell deletion showed significant intestinal damage than WT mice, and the intestinal inflammation was exacerbated in MafK Tg mice (Figure 1G).

**Figure 2:**
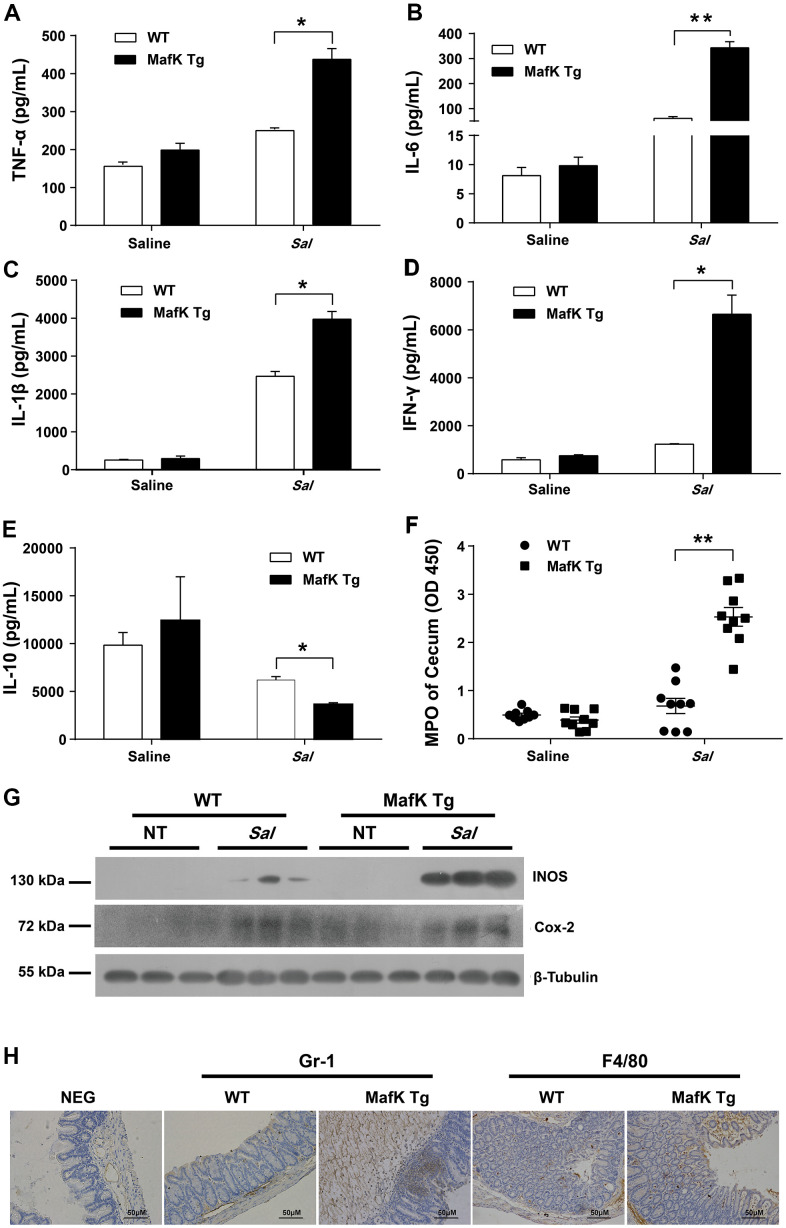
**MafK leads to abnormal inflammatory responses.** Streptomycin-pretreated WT and MafK mice were orally infected with *Salmonella* (n = 9 each group) for 48 h. The homogenate supernatant of cecum was detected for concentrations of indicated cytokines by ELISA. (**A**) TNF-α, (**B**) IL-6, (**C**) IL-1 β, (**D**) IFN-γ, (**E**) IL-10. (**F**) The homogenate supernatant of the cecum was also used to determine the activity of MPO. (**G**) Representative cecum tissue lysates were analyzed for iNOS and Cox-2 by western blotting. GAPDH was used as a loading control. (**H**) Representative immunohistochemical staining of Gr-1 (a neutrophil marker) and F4/80 (a macrophagocyte marker) were performed in the cecal sections. All data are shown as the mean ± SEM. Student’s t test was performed. Statistical significance is indicated by *p < 0.05, **p < 0.01.

WT and MafK Tg mice were infected with *Salmonella* for 2 days to explain the involvement of MafK in intestinal colitis limitation by evaluated the production of proinflammatory mediators in the ceca. Compared with WT mice, the cecum of MafK Tg mice contained considerably more proinflammatory cytokines, TNF-α, IL-6, IL-1β, and INF-γ were all included (Figure 2A–2D). TNF-α, IL-6, and INF-γ’s Levels in the serum of MafK Tg mice were all significantly upregulated compared to WT mice following a 2 day infection with *Salmonella* (Supplementary Figure 1). Comparatively, when compared to WT mice, TNF-α, IL-6, and INF-γ of MafK Tg mice were increased significantly following *Salmonella* infection for 2 days in the colon (Supplementary Figure 3B–3D). The anti-inflammatory cytokine IL-10 was found to be significantly reduced in MafK Tg mice compared to WT mice (Figure 2E). In addition, neutrophil accumulation was elevated by myeloperoxidase (MPO) activity in MafK Tg mice (Figure 2F). MafK Tg mice had increased levels of the inflammatory enzymes Cox-2 and iNOS, both of which have been linked to colon inflammation and cancer (Figure 2G). Additionally, on Day 2 p.i. MafK Tg and WT mice accumulated neutrophils and macrophages (Figure 2H).

Overall, MafK Tg mice presented extensive intestinal epithelium damage in the cecum and colon with significant inflammation. That indicated MafK is associated with susceptibility to *Salmonella* mucosal infection.

### MafK increased the dissemination of *Salmonella*

**Figure 3:**
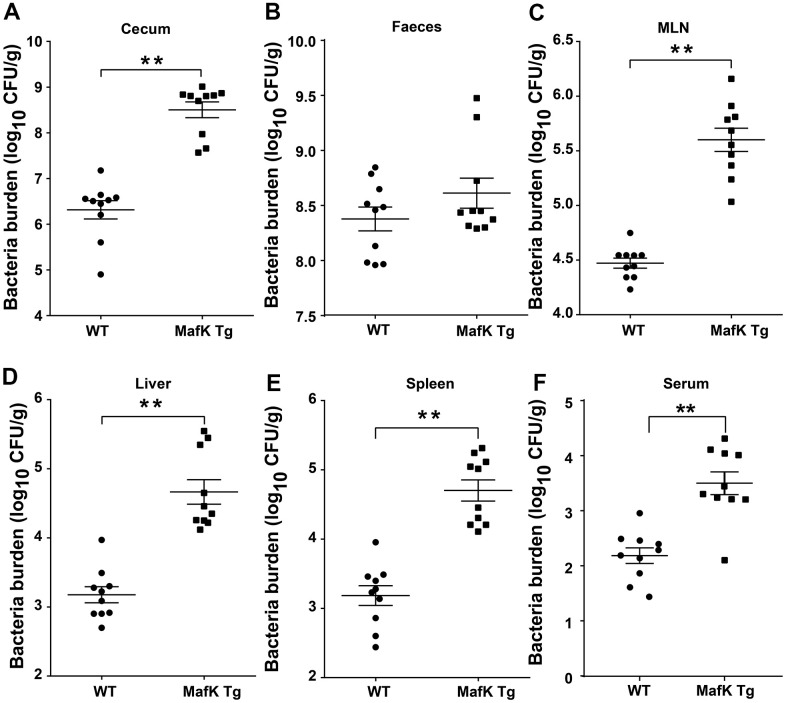
**MafK enhances *Salmonella* invasion and dissemination.** Streptomycin-pretreated WT and MafK Tg mice were orally infected with *Salmonella* (n = 10 each group) for 48 h. Bacterial loads in the cecum (**A**), faeces (**B**), MLN (**C**), liver (**D**), spleen (**E**), and serum (**F**) were detected. All data are shown as the mean ± SEM. Student’s t test was performed. Statistical significance is indicated by *p < 0.05, **p < 0.01.

On the basis of the aforementioned findings, we hypothesized that the increased pathology observed in MafK Tg mice after *Salmonella* infection was caused by greater colonization levels. *Salmonella* burdens in the cecum and feces of MafK Tg mice were dramatically increased (Figure 3A, 3B), suggesting that MafK promotes *Salmonella* colonization in the intestinal tract. Moreover, *Salmonella* levels were significantly increased in MafK Tg mice’s mesenteric lymph nodes (MLNs), liver, spleen, and serum when compared to WT mice (Figure 3C–3F), implying what MafK Tg mice failed to control *Salmonella* translocation. Thus, these results suggested that MafK was associated with susceptibility to *Salmonella* mucosal infection, which resulted in quicker *Salmonella* dissemination.

### MafK has a mild effect on the expression of tight junction protein

**Figure 4:**
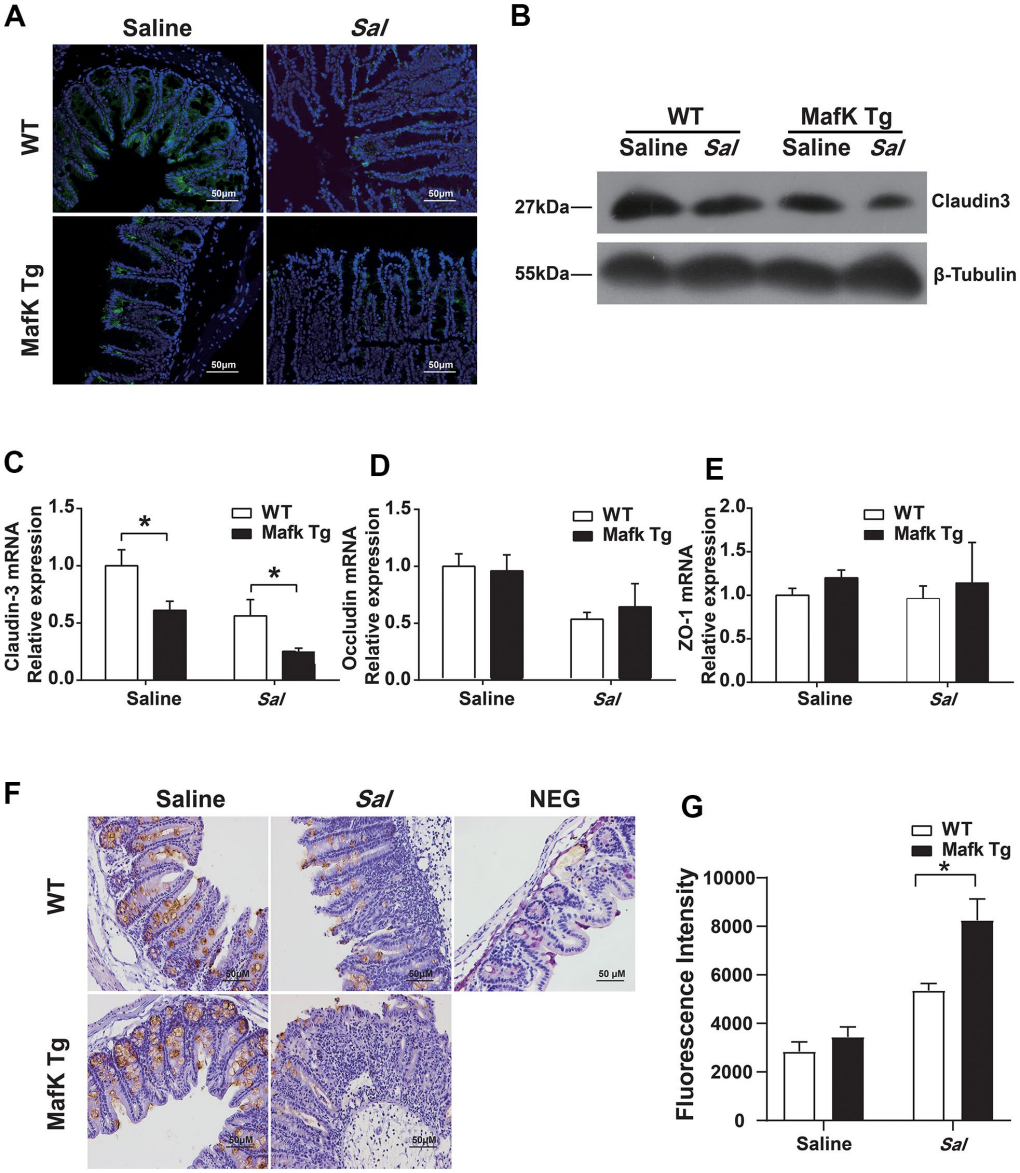
**MafK increases intestinal barrier damage during *Salmonella* infection.** Streptomycin-pretreated WT and MafK Tg mice were orally infected with *Salmonella* (n = 10 each group) for 48 h. Claudin3 expression was examined in cecum sections by (**A**) immunofluorescence and (**B**) western blotting. The mRNA levels of (**C**) claudin-3, (**D**) occludin and (**E**) ZO-1 in the cecum were examined by real-time PCR. The data were normalized to GAPDH and are shown as the fold increase in mRNA. (**F**) Immunohistochemistry staining for mucin 2. (**G**) Intestinal permeability was measured using FITC-dextran. *p < 0.05, **p < 0.01.

The increased bacterial colonization and dissemination indicated a fragile barrier in MafK Tg mice. Tight junctions seal the gap between the cells, and contribute to maintaining the integrity of the gastrointestinal epithelium and against microbial infection as a permeable barrier. Claudin, occludin and zonula occludens (ZO)-1 as scaffolding proteins constitute the tight junctions. Therefore, we started to investigate MafK’s effects on tight junctions. When compared to WT mice, the expression and the mRNA basal level of claudin3 (Figure 4A–4C) in MafK Tg mice were decreased, whereas the mRNA of occludin (Figure 4D) and ZO-1 (Figure 4E) didn’t show difference in basal level. The results were consistent with claudin3, occludin and ZO-1 mRNA levels (Supplementary Figure 2A–2C) in MafK-overexpressing Caco-2 cells *in vitro*. Following *Salmonella* administered orally, claudin3 and occludin levels were decreased in WT mice, while the reduction of claudin3 or occludin was not obvious in MafK Tg mice, this could be related to the reduced amount of tight junction protein expression already present. Mucin 2, the mucus layer's primary component was additionally examined. No difference was found between naïve WT mice and MafK Tg mice; whereas, following *Salmonella* infection, the amount of mucin 2 decreased in MafK Tg mice (Figure 4F). To further determine whether MafK overexpression increases the sensitivity to *Salmonella*-induced colitis associated with intestinal barrier function, the fluorescent tracer FITC-dextran was used to assess the paracellular intestinal permeability. On Day 2 p.i., the serum of MafK Tg mice contained substantially more FITC-dextran than that of WT animals mice (Figure 4G), indicating that the barrier function of MafK Tg mice was disrupted. Based on these studies, MafK was found to have a detrimental influence on the function of the intestinal barrier, MafK overexpression led to the deregulation of the claudin-3 protein, making *Salmonella* much easier to disorganize the epithelial barrier.

Collectively, these data suggested that the failure of MafK Tg mice to protect against *Salmonella* infection might result from the fragile tight junction barrier function.

### MafK increased epithelial cell apoptosis in response to oral *Salmonella* infection

**Figure 5:**
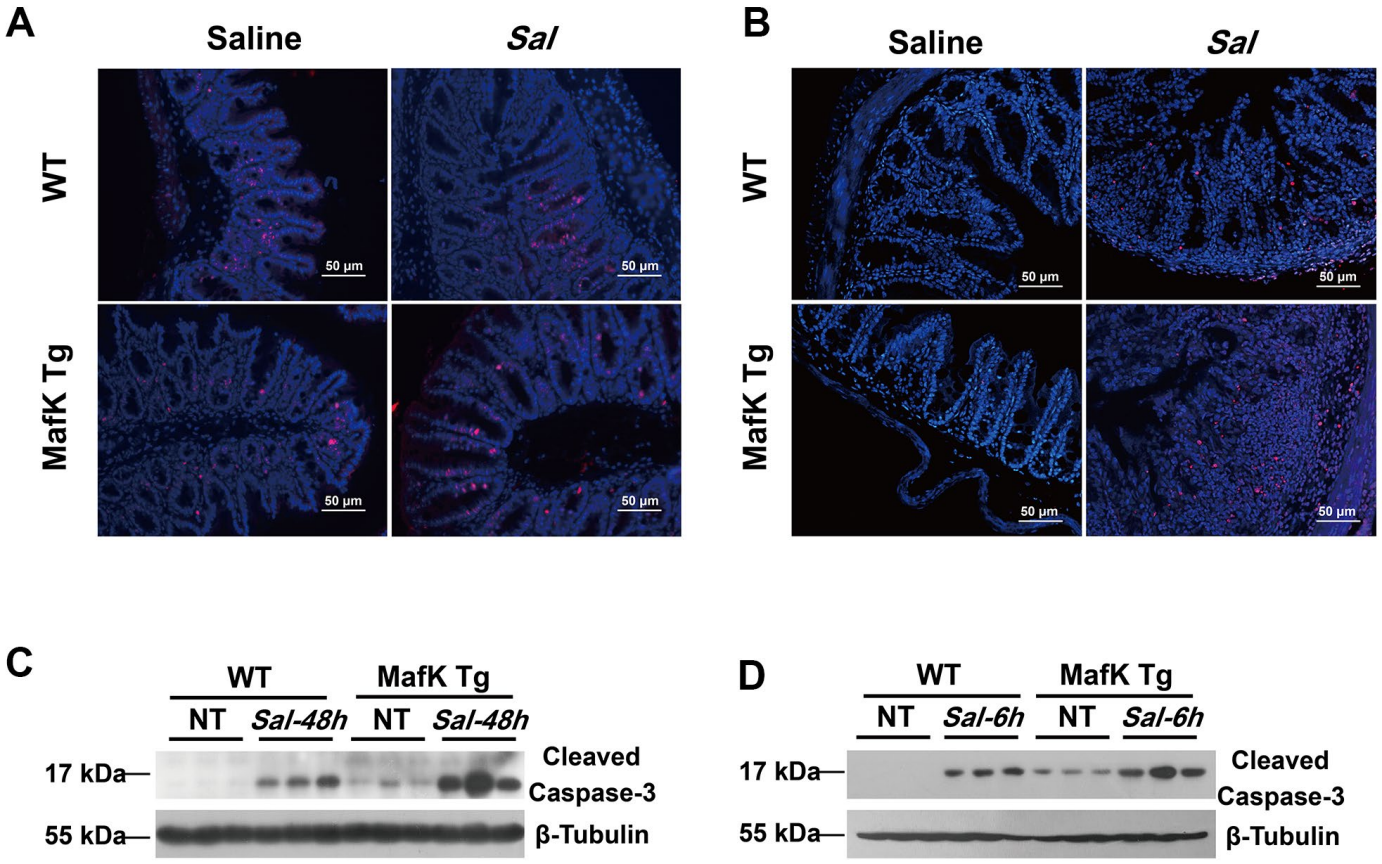
**MafK promotes epithelial cell apoptosis following *Salmonella* oral challenge.** (**A**) Immunofluorescence was performed on cecum sections derived from WT and MafK Tg mice on Day 2 post *Salmonella* oral infection to examine proliferating cells (Ki-67). (**B**) Cell death was identified in the cecum by TUNEL staining. Levels of cleaved caspase-3 expression in cecum lysates on Day 2 (**C**) and 6 h (**D**) after *Salmonella* oral infection were determined by western blotting.

Maintaining tissue homeostasis is crucial during infection and inflammation. The regulation of cell turnover comprised of both proliferation and apoptosis, makes a contribution to the preservation of intestinal homeostasis. The intestinal barrier function was negatively affects by the loss of intestinal. To demonstrate the differential in cell proliferation and apoptosis that may account for the increased sensitivity to *Salmonella* colitis and the increased *Salmonella* dissemination in MafK Tg mice, cell proliferation was examined by immunofluorescence staining of proliferating cells nuclear antigen Ki-67. According to the findings, epithelial cell proliferation was unaffected by MafK in naïve mice. The level of epithelial cell proliferation, on the other hand, increased significantly after *Salmonella* oral administration in MafK Tg mice (Figure 5A). Following, TUNEL staining showed that MafK Tg mice had greatly increased cell death in the epithelium on Day 2 p.i., while a little increase of cell death in the epithelium was found in WT mice (Figure 5B). Additionally, cleaved caspase-3 as effector molecule of apoptosis was confirmed by western blotting in the cecum. Similar to the TUNEL staining results, cleaved caspase-3 was higher in cecum of MafK Tg mice than WT mice following *Salmonella* infection for 2 days (Figure 5C); however, this phenomenon might have been cause by overt tissue damage. Therefore, the level of apoptotic signaling molecules in the cecum was examined at an early stage of infection with no obvious inflammation or histopathology. Remarkably, MafK Tg mice presented a higher level of cleaved caspase-3 at 6 h p.i. (Figure 5D). Collectively, these data indicated that MafK promotes epithelial cell apoptosis when challenged with *Salmonella*.

### MafK induces enhanced epithelial cell apoptosis

**Figure 6:**
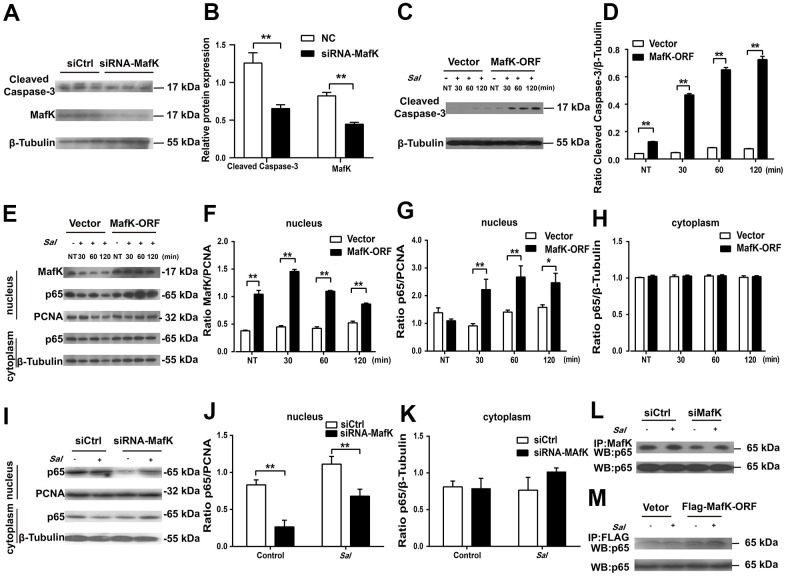
**MafK is involved in the activation of caspase-3 and p65.** (**A**, **B**) Levels of cleaved caspase-3 in siRNA MafK Caco-2 cells and control cells. (**C**, **D**) Levels of cleaved caspase-3 in MafK-overexpressing Caco-2 cells and control cells infected with *Salmonella*. (**E**–**H**) Levels of MafK and p65 in MafK-overexpressing Caco-2 cells and control cells infected with *Salmonella*. (**I**–**K**) Levels of p65 in siRNA MafK Caco-2 cells and control cells infected with *Salmonella*. Levels of p65 in MafK siRNA Caco-2 cells (**L**) and MafK-overexpressing Caco-2 cells (**M**). *p < 0.05, **p < 0.01.

Based on the observation we found *in vivo*, MafK has a promoting effect on epithelial cell apoptosis, so the effect was further studied *in vitro*. A previous report showed that the death of IECs triggered severe chronic inflammatory pathologies [20]. To elaborate the mechanism by which MafK promotes epithelial cell apoptosis and contributes to increased susceptibility to *Salmonella* infection, MafK was interrupted or overexpressed in Caco-2 cells, and apoptosis signaling molecules were examined. The level of cleaved caspase-3 in MafK-interrupted Caco-2 cells was lower than the control cells, indicating a positive effect of MafK on apoptosis (Figure 6A, 6B). The association between MafK and cleaved caspase-3 was further confirmed in MafK-overexpressing Caco-2 cells*.* Cleaved caspase-3’s content in MafK-overexpressing Caco-2 cells was increased than the control cells. Following infection by *Salmonella*, Caco-2 cells overexpressing MafK had much higher levels of cleaved caspase-3 compared to the results obtained from the control cells (Figure 6C, 6D). These findings imply that MafK could promote caspase-3 activation.

MafK can interact with different mediators to execute different biological functions. Previous reports indicated that MafK mediates p65 acetylation and participates in oxidative stress and inflammation [21]. Furthermore, NF-κB signaling could induce caspase-3-mediated apoptosis in osteoclasts [22]. To determine whether there was a correlation between MafK and p65, the levels of p65 were measured in the nucleus of MafK-overexpressing Caco-2 cells and control cells. In the nucleus, the content of p65 presented similarity between MafK-overexpressing Caco-2 cells and the control cells. In response to the *Salmonella* infection, the levels of p65 in the nucleus of MafK-overexpressing Caco-2 cells were significant increased than those in control cells. Similarly, the level of MafK also increased gradually when challenged with *Salmonella.* However, few changes were found in the control cells regardless of the expression of MafK or the nuclear entry of p65. After *Salmonella* infection in MafK-overexpressing Caco-2 cells and control cells, the levels of p65 in the cytoplasm were not different (Figure 6E–6H). These results indicated that MafK can accelerate the nuclear entry of p65 during *Salmonella* infection. Additionally, in siRNA MafK Caco-2 cells and control cells, the levels of p65 in the cytoplasm and nucleus were examined. A similar regulation effect was found. The levels of p65 in the nucleus in MafK-silenced Caco-2 cells were reduced compared with those in the control cells, while p65’s content were not affected in the cytoplasm, indicating a promoting effect of MafK on p65 (Figure 6I–6K). The subsequent pull-down study proved that MafK could interact with p65 directly (Figure 6L, 6M). According to these results, MafK might promote cleaved caspase-3 expression through increasing p65 entry nucleus.

Collectively, these results indicated that MafK could participate in accelerating the invasion of *Salmonella* pathogenesis in the mouse intestine by increasing caspase-3 activation.

## DISCUSSION

As a small Maf protein (MafK, MafG or MafF), MafK can form homodimers to repress transcription or form heterodimers with other transcription factors that determines whether they repress or stimulate transcription [23]. In the Japanese population, 226 patients with UC and 748 subjects as control were examined, and the 226 UC patients showed an increase in MafK expression, indicating that the high levels of MafK could aggravate the progress and exacerbation of UC [24]. Nevertheless, the biological implications of MafK in the intestine remain unknown. To better clarify how MafK is associated with intestinal function, a *Salmonella* colitis model was used in this study. We found that apoptosis induced by MafK was enhanced in epithelial cells and contributed to the increased sensitivity to *Salmonella*-induced colitis and uncontrollable bacteria dissemination in MafK Tg mice.

The pathophysiology of IBD is aggravated by the dysregulation of the intestinal immune response. To investigate whether the local immune response could be activate by MafK in the gastrointestinal tract, a dose of 5×10^7^ CFU *Salmonella* was given orally to the mice. The results showed that MafK has a negative effect on gut homeostasis. MafK Tg mice had increased production of proinflammatory mediators, making them more sensitive to *Salmonella* mucosal infection and sustained more intestinal damage.

It was revealed that MafK is essential in susceptibility to the inflammatory response and the disruption of intestinal immune in MafK Tg mice due to the uncontrolled inflammatory response and extravagant intestinal damage. Therefore, it is necessary to examine the mechanisms behind the impairment of intestinal immunological homeostasis in MafK Tg mice.

*Salmonella* reaches the intestinal epithelium following orally administration and eventually arrives colonize systemic sites from the gastrointestinal tract. It is beneficial for the dissemination of microbes in the host to disrupt the intestinal barrier. Therefore, the dysfunctional epithelial barrier could cause increased bacterial dissemination in MafK Tg mice. Intestinal epithelium tight junctions are important for defending against bacterial pathogen invasion [25]. Thus, the effect of MafK on tight junctions were examined. Our studies showed only a mild decrease in the expression of claudin 3 in naïve MafK Tg mice, which might have contributed to the increased susceptibility to *Salmonella* infection. Occludin and ZO-1, in contrast to claudin 3, were unaffected in naïve MafK Tg mice. Mucins in the cecum are also important in intestinal defense and homeostasis [26]. Although there was a decrease in mucin 2 expression following *Salmonella* infection in MafK Tg mice, it might have been caused by overt tissue damage leading to an increased loss of goblet cells. In IBD, leaky diarrhea, epithelium-derived cancers, loss of tight junction integrity and function is commonly recognized [27]. No evidence of spontaneous inflammation was identified in MafK Tg mice with a defect of claudin 3 expression. Based on this finding, it indicates that intestinal barrier disruption alone is not sufficient to generate spontaneous intestinal inflammation. A compensating mechanism could assist to maintain MafK Tg mice’s homeostasis when uninfected. Due to the vulnerable intestinal barrier, MafK Tg mice are more susceptible to be infected in a hostile environment.

Cell proliferation control is critical to histological healing [28]. Disorder of intestinal epithelial cell proliferation and death will cause the host to be continuously exposed to potentially inflammatory stimulation [29]. Compared with WT mice, MafK Tg mice did not respond normally to *Salmonella* challenge, which was characterized by increased epithelial cell apoptosis. These results suggest that MafK disturbs the maintenance of intestinal apoptosis homeostasis. However, the aberrant epithelial apoptosis in MafK Tg mice is probably a result of exacerbated inflammation on Day 2 p.i. To explore this possibility, epithelial cell apoptosis was further examined at an early stage with no obvious histopathology or inflammation. MafK Tg mice had more apoptotic cells than WT mice following *Salmonella* infection for 6 h. Similarly, the levels of cleaved caspase3 also increased in MafK-overexpressing cells and decreased in MafK-interrupted Caco-2 cells. These data indicate that MafK can induce epithelial cell apoptosis by promoting cleaved caspase-3 expression. Following *Salmonella* infection, MafK induces epithelial cells to progress to quicker apoptosis, which results in a fragile intestinal barrier that is more easily penetrated by microbes. A subsequent study found that MafK act as a modulator of NF-κB promoting the nuclear entry of p65. The promoting effect of MafK on p65 is consistent with cleaved caspase-3, indicating a possible association between MafK/p65 and cleaved caspase-3 (Supplementary Figure 4). This is similar to a previous report showing that the NF-κB signaling pathway promotes caspase-3-mediated apoptosis [22]. Our study revealed that MafK promotes NF-κB activation and apoptosis in intestinal epithelial cells, resulting in a rapid cell death and inflammation induction. The disorder of MafK function might increase the process of inflammatory diseases, such as UC patients with MafK overexpression [24]. Inhibition of MafK signaling will alleviate inflammation induction and cell apoptosis. Therefore, the development of MafK inhibitor can be used as a treatment strategy for intestinal disease. Many inflammatory chronic diseases, such as IBD, has an increased NF-κB activation. The development of MafK inhibitors will inhibit the function of NF-κB in a specific pathway. And MafK inhibitors might be used as a caspase-3 inhibitor for therapeutic utility to caspase-3-related pathological conditions. More studies are needed to find the effective MafK inhibitors and the application of MafK inhibitors.

## CONCLUSIONS

In conclusion, this study reveals that MafK participates in *Salmonella* pathogenesis acceleration by increasing caspase-3 activation. And our findings provide a new insight into the possible role of MafK in regulating epithelial cell apoptosis which facilitates *Salmonella* invasion.

## Supplementary Material

Supplementary Figures
