# Supplementary material for: MafK accelerates Salmonella mucosal infection through caspase-3 activation
Source: Aging (Albany NY). 2022 Mar 8;14(5):2287–303. doi: 10.18632/aging.203938 (PMC8954977; doi:10.18632/aging.203938)
Supplement: Supplementary Figures [file aging-14-203938-s001.pdf]

SUPPLEMENTARY FIGURES

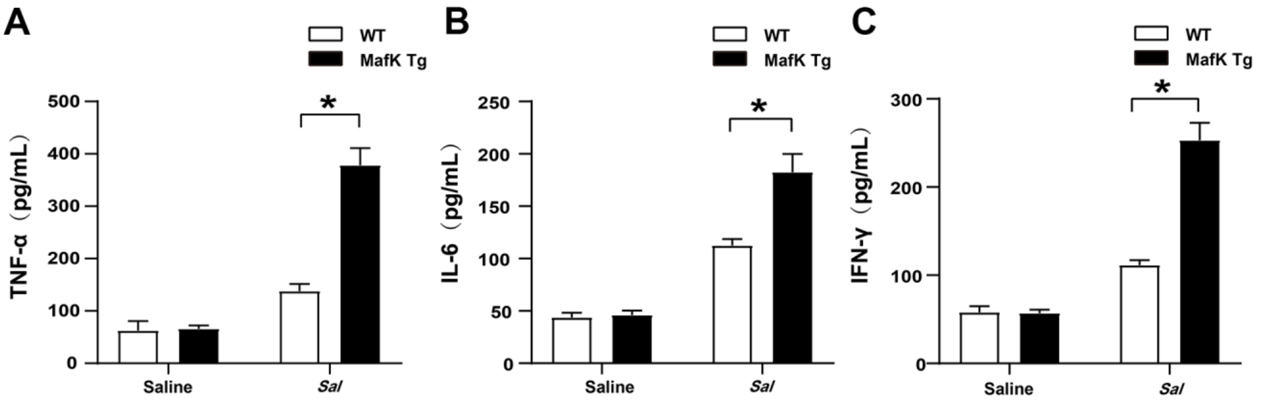

Supplementary Figure 1. Cytokines of serum in WT and MafK Tg mice. Streptomycin-pretreated WT and MafK mice were orally infected with *Salmonella* (n = 5 each group) for 48 h. The cytokines of serum was detected for concentrations by ELISA. (**A**) TNF-α, (**B**) IL-6, (**C**) IFN-γ. All data are shown as mean ± SEM. Student's t-test was performed. Statistical significance is indicated by *$p < 0.05$, **$p < 0.01$.

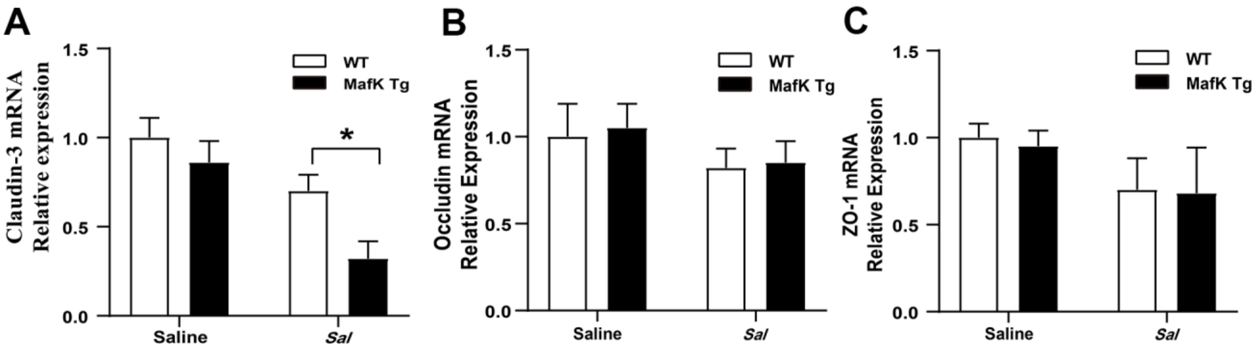

Supplementary Figure 2. Tight junction proteins were examined in MafK-overexpressing system by RT-PCR. The mRNA levels of (**A**) claudin3, (**B**) occludin and (**C**) ZO-1 in MafK-overexpressing Caco-2 cells and control cells were examined by real-time PCR. The data were normalized to GAPDH and are showed as the fold increase in mRNA. *$p < 0.05$, **$p < 0.01$.

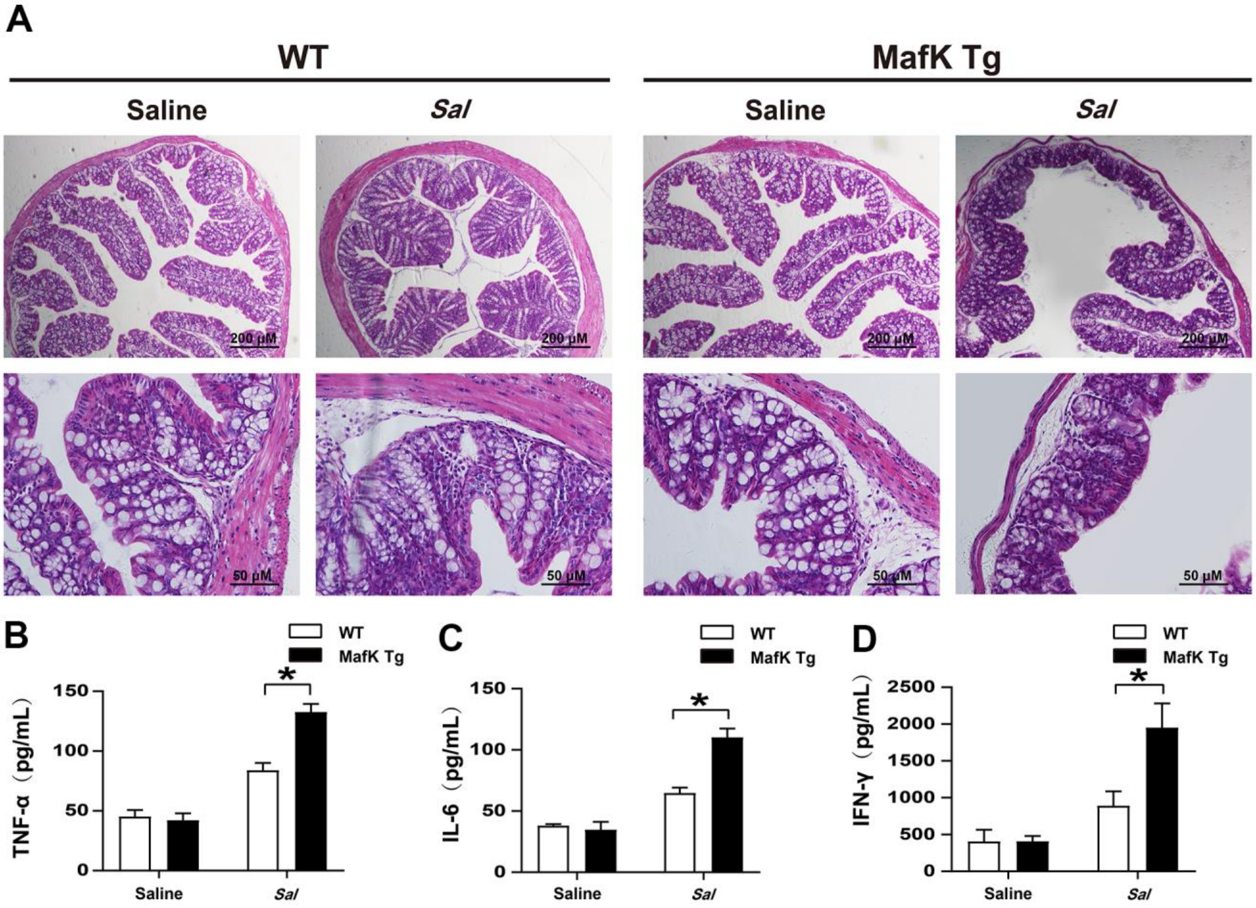

Supplementary Figure 3. *Salmonella* infection induces inflammation in colon. Streptomycin-pretreated WT and MafK mice were orally infected with *Salmonella* (n = 5 each group) for 48 h. (**A**) Representative H&E staining of colon. The homogenate supernatant of colon was detected for concentrations of indicated cytokines by ELISA. (**B**) TNF-α, (**C**) IL-6, (**D**) IFN-γ, All data are shown as mean ± SEM. Student's t-test was performed. Statistical significance is indicated by *$p < 0.05$, **$p < 0.01$.

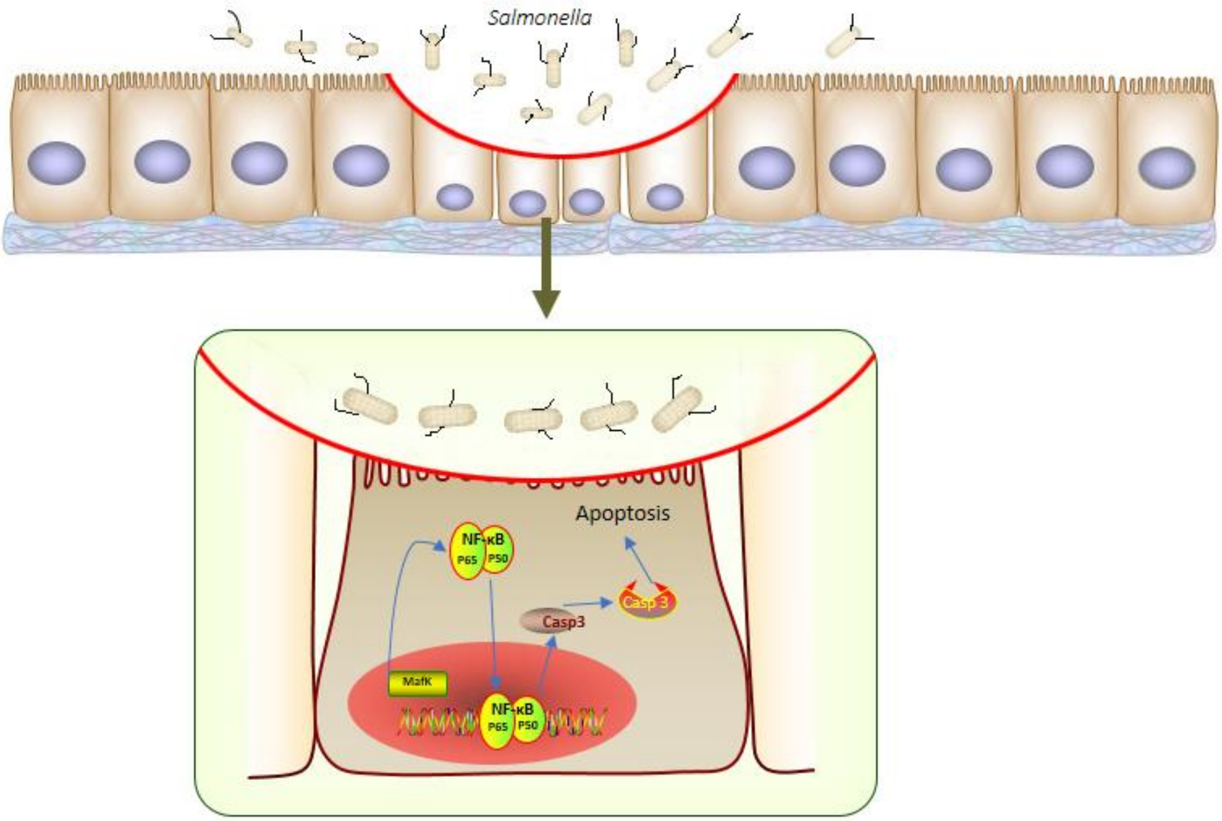

Supplementary Figure 4. Schematic depiction of MafK function and regulation in *Salmonella* mucosal infection. MafK might accelerate *Salmonella* mucosal infection through increasing intestinal epithelial cell apoptosis by increasing p65 entering the nucleus and then inducing caspase-3 cleavage to break down the intestinal barrier.
